# Understanding Problematic Smartphone and Social Media Use Among Adults in France: Cross-Sectional Survey Study

**DOI:** 10.2196/63431

**Published:** 2025-03-06

**Authors:** Laurent Karila, Nathaniel Scher, Clement Draghi, Delphine Lichte, Ilan Darmon, Hanene Boudabous, Hanah Lamallem, Olivier Bauduceau, Marc Bollet, Alain Toledano

**Affiliations:** 1 Center for Teaching, Research, and Treatment of Addictions, Paul Brousse University Hospital, Paris Saclay University - PSYCOMADD Research Unit, Villejuif, France; 2 Rafael Institute, Integrative Medicine Center, 3 boulevard Bineau, Levallois-Perret, 92300, France, 33 658312182; 3 Hartmann Oncology Radiotherapy Group, Hartmann Radiotherapy Center, Levallois-Perret, France; 4 PRO BTP Group, Paris, France; 5 Department of Integrative Medicine, Conservatoire National des Arts et Metiers, Paris, France

**Keywords:** problematic smartphone use, digital mental health, compulsive behavior, social network addiction, nomophobia, phubbing, screen time effects, public health interventions, cross-sectional, French, smartphone use, social media, France, mobile phone

## Abstract

**Background:**

The Evaluation of Digital Addiction (EVADD) study investigates problematic smartphone use in the digital age, as global smartphone users reached 55.88 million in France in 2023. With increased screen time from digital devices, especially smartphones, the study highlights adult use issues and associated risks.

**Objective:**

The aim of the study is to assess the prevalence of compulsive smartphone use among French adults and identify patterns of problematic behaviors, including their impact on daily activities, sleep, and safety.

**Methods:**

The EVADD study used a cross-sectional, nonprobability sample design, conducted from May 3 to June 5, 2023. Participants were recruited through the French mutual insurance company PRO-BTP. Data were collected anonymously via a digital questionnaire, including the Smartphone Compulsive Use Test, capturing information on social network engagement, device ownership, and daily screen use.

**Results:**

A total of 21,244 adults (average age 53, SD 15 years; 9844 female participants) participated. Among 21,244 participants, 8025 of 12,034 (66.7%) respondents exhibited compulsive smartphone use (*P*<.001). Additionally, 7,020 (36.7%) participants scored ≥8 on the Smartphone Compulsion Test, indicating addiction. Younger participants (18‐39 years) were significantly more likely to show signs of addiction (2504/4394, 57%; odds ratio 2.5, 95% CI 1.9‐3.2) compared to participants aged ≥60 years. Problematic behaviors included unsafe smartphone use while driving (5736/12,953, 44.3%), frequent smartphone use before bedtime (9136/21,244, 43%), and using smartphones in the bathroom (7659/21,244, 36.1%). Sleep disturbances and risky behaviors correlated strongly with higher compulsion scores (*P*<.01).

**Conclusions:**

The EVADD study highlights the complex relationship between adults and smartphones, revealing the prevalence of compulsive behaviors and their impact on daily life, sleep, and safety. These findings emphasize the need for public awareness campaigns, preventive strategies, and therapeutic interventions to mitigate health risks associated with excessive smartphone use.

## Introduction

### Background and Digital Addiction

The term “digital” encompasses the use of digital technology for communication through intangible devices. This includes activities such as browsing social networks, playing video games, or engaging in digital gambling using computers, smartphones, and tablets.

The proliferation of screens is a relatively recent phenomenon, with the number of smartphone users in France estimated to have reached 55.88 million in 2023, marking a doubling since 2013 [1]. Screen selection is often dictated by various contexts, including sedentary lifestyles, mobility, and personal or professional use. Consequently, the time spent on screens, known as screen time, has witnessed a steady increase over the years. This trend has contributed to a notable rise in the prevalence of excessive screen behavior among individuals across age groups, including children, adolescents, and adults [2].

Problematic smartphone use is characterized by 3 primary consumption problems: addictive consumption habits (eg, symptoms of addiction), antisocial consumption patterns (eg, prohibited use), and adverse use patterns (eg, phone use while driving and going to the toilets or to bed with a smartphone), leading to a negative impact on academic, professional, or social functioning. This negative influence can manifest in disrupted interpersonal relationships, social withdrawal due to limited face-to-face contact, and an increased preference for solitary activities over group interactions. While smartphone addiction does not equate to substance or gambling addiction, screens can indeed foster addictive processes through activities like video gaming, social networking, and engaging in sexual content [3].

The widespread adoption of smartphones has given rise to a new digital clinical terminology. “Nomophobia,” or “no-mobile-phone-phobia,” refers to the compulsive and time-intensive use of smartphones, coupled with anxiety when the device is unavailable. It encompasses symptoms such as a constant need for availability, a preference for mobile communication over face-to-face interaction, and financial challenges related to excessive mobile use. “Ringxiety,” a specific manifestation of nomophobia rather than a standalone construct, describes the sensation of hearing phantom phone rings or notifications. Meanwhile, “fear of missing out” (FoMO) drives attentional distraction, as individuals strive to stay connected and engaged with the positive digital experiences portrayed in others’ content [4]. Both nomophobia and FoMO reinforce the need for constant connection, perpetuating excessive smartphone use. “Phubbing,” a blend of “phone” and “snubbing,” refers to ignoring others by focusing on one’s phone during social interactions [5]. “Athazagoraphobia” refers to the fear of being forgotten or ignored, often manifesting as anxiety when messages, posts, or comments go unnoticed, while “smombie,” a contraction of “smartphone zombie,” describes individuals walking while absorbed in their phones, oblivious to their surroundings. These behaviors often result in compulsive checking of messages or social networks [6].

Research has highlighted the fine line between habitual, nonproblematic use of social networks and potentially addictive behavior. Users exhibiting symptoms and consequences akin to substance addiction, such as salience, mood changes, tolerance, craving, withdrawal, and relapse, may indeed be addicted to social networks. Our objective is not to pathologize everyday behaviors excessively but to gather valuable insights that can help prevent risky behaviors and provide support for those in need of therapeutic intervention [7].

These digital behaviors have thus become a growing public health concern. Moreover, several recent studies conducted after the most acute phases of the COVID-19 pandemic have highlighted evolving digital use patterns. These include increased reliance on smartphones for communication, remote work, entertainment, and social networking, potentially reflecting longer-term shifts in digital habits [8-10]. Such postpandemic data provide a more contemporary context for understanding current problematic smartphone use trends.

### Study Objectives

The objective of this study is to evaluate the extent and patterns of problematic smartphone and social media use among adults in France using validated assessment tools. Specifically, we aim to identify the prevalence of compulsive behaviors, their associations with demographic factors, and the potential health and social consequences.

## Methods

### Ethical Considerations

This study was conducted in accordance with the ethical principles of the Declaration of Helsinki and the French Public Health Code (Article L.1123-7). The research protocol was reviewed and approved by the Comité de Protection des Personnes Sud-Est I (reference 2023-A00312-43, national number 23.00519.000257). The approval was granted on March 6, 2023, and remains valid for 2 years. Participants were provided with detailed information regarding the study objectives, data collection process, confidentiality measures, and their rights, including the ability to withdraw at any time without consequences. Written informed consent was obtained from all participants before their inclusion in the study. To ensure compliance with the General Data Protection Regulation and French data protection laws, the study was designed to protect participant confidentiality. Data were anonymized and securely stored. Participants had access to the data protection officer for any inquiries or concerns regarding their data rights. No financial or material compensation was provided to participants in this study.

### Study Design and Participant Characteristics

The Evaluation of Digital Addiction (EVADD) study used a cross-sectional, nonprobability sample design from May 3 to June 5, 2023. The use of nonprobability sampling was dictated by the practical constraints of recruiting participants through the PRO-BTP network, which provided access to a large population of insured members. To ensure comprehensive and transparent reporting of our web-based data collection, we have adhered to the CHERRIES (Checklist for Reporting Results of Internet E-Surveys) guidelines.

A total of 800,000 members of the French mutual insurance company PRO-BTP were invited to participate via email. The email provided information about the survey’s objectives, contents, and confidentiality protections, along with access to a completely anonymous digital questionnaire through a provided link. Upon clicking the link, participants were automatically redirected to a web platform hosted by the researchers, where they could complete the questionnaire anonymously. No personal data such as email or IP addresses were collected. Participants were required to be at least 18 years of age and proficient in French; no other eligibility criteria were applied. The study followed the CROSS (Checklist for Reporting of Survey Studies) for reporting survey studies, as recommended by the Enhancing the Quality and Transparency of Health Research network. Considering an expected response rate of 1%‐5% among PRO-BTP members, corresponding to 8000 to 40,000 responses, a sample size of 16,302 participants was determined necessary to achieve a reliable representation of PRO-BTP members with a 99% CI and a 1% error margin.

Participant characteristics collected included age, sex, socioprofessional category, education level, and family status. Age was categorized into 3 groups: 18‐39, 40‐59, and ≥60 years. Socioprofessional categories were classified into 8 groups based on the French national classification system.

### Measurement Tools: Smartphone Compulsion Scores

Data were obtained through a combination of a standardized test and author-formulated questions. The primary outcome measure was the degree of participants’ smartphone compulsive use, assessed using the Smartphone Compulsive Use Test score [11], consisting of 15 yes or no questions. Scores indicated normal behavior (1‐2 yes), leaning toward excessive use without problems (3‐4 yes), problematic or compulsive use (5 or above), and obvious behavioral addiction (8 or higher).

The Smartphone Compulsion Test (SCT) was selected, as it broadly evaluates the clinical aspects of problematic smartphone use and its consequences. While no validated tools currently exist in French for this purpose, the SCT has been extensively used in our clinical practice. The trends observed in this study will serve as a basis for formal psychometric validation of this tool in the near future.

The SCT was administered to participants who reported owning and regularly using a smartphone. Respondents who did not own a smartphone or did not report regular use were excluded from this portion of the survey. This criterion ensured the relevance of the questions to the participants’ experiences.

### Measurement Tools: Problematic Smartphone Behaviors

A total of 4 yes-no questions assessed smartphone use while driving. In total, 10 questions evaluated nomophobia, while 1 question each assessed athazagoraphobia, phubbing, and smombie behavior. Additionally, participants were asked how frequently they took their smartphone or tablet to the toilet or bed, with response options of never, rarely, often, and always.

### Measurement Tools: Social Media Use Patterns

All participants were asked whether they used social media networks. Those who confirmed using at least 1 social network were subsequently directed to questions about their use patterns.

The 5 addiction-related questions were then administered to a subset of participants (approximately 4800) who reported frequent use of social media and behaviors indicative of potential problematic use, as identified through initial screening questions. The 5 yes-no questions related to the activity on networks, and 5 others, to be answered by never, rarely, often, and always, related to a possible problem with network use.

Participants reported the number and types of electronic devices they owned (smartphone, tablet, laptop, desktop, and game console) and their primary use (social network, messaging, SMS text messages, emails, television series seasons, video games, and digital gambling). Daily leisure time spent on these devices was also estimated.

The questionnaire was pretested by a small group of researchers to ensure clarity, coherence, and technical functionality before wide distribution. No forced responses or completeness checks were implemented, allowing participants to proceed without answering all questions. Respondents were not able to return to previous pages to modify their answers once they advanced. Additionally, no statistical weighting or adjustments were performed to correct for the potential nonrepresentativeness of the sample.

### Statistical Analysis

Descriptive statistical analyses were performed using R software (version 4.2; R Foundation for Statistical Computing). Descriptive statistical analyses were conducted to summarize participant characteristics and responses. Continuous variables were analyzed using nonparametric tests (eg, Kruskal-Wallis and Mann-Whitney-Wilcoxon) due to the nonnormal distribution of data, which was confirmed through the Shapiro-Wilk test. Categorical variables were analyzed using chi-square tests, appropriate for the distribution and nature of the data. Comparisons of median compulsion scores across groups used nonparametric methods due to the ordinal nature of the data and the violation of normality assumptions. Homogeneity of variances was assessed using the Levene test, and where assumptions were violated, appropriate adjustments were made (eg, robust statistical methods).

Categorical variables were presented as frequencies and percentages, while continuous variables were described using means and SDs. The number of missing data for each parameter was reported. Median compulsion scores were compared across age groups and digital activities using appropriate statistical tests such as Kruskal-Wallis or Mann-Whitney-Wilcoxon tests.

## Results

### Participant Characteristics

Overall, 21,244 (2.7% of the solicited panel) persons responded to all or parts of the questionnaire. Their overall characteristics are shown in Table 1. Their mean age was 53 (SD 15 years), and 9844 (46.3%) were female participants. The majority of participants were retirees or employees, and 14,293 (72%) were married or lived in a couple. More than 10,622 (50%) participants had 2 or 3 devices, mainly smartphones and laptops. The favorite device was the smartphone, and 14,871 (70%) participants admitted that they could no longer do without it.

**Table 1.** Participant characteristics.

Responder characteristics	Values (N=21,244)	OR^a^ (95% CI)	Answers, n
Age (years), mean (SD)	53 (15)	—^b^	19,720
**Age group (years), n (%)**			—
18‐39	4394 (22)	0.28 (0.27-0.29)	
40‐59	7650 (39)	0.64 (0.62-0.66)	
≥60	7660 (39)	0.64 (0.62-0.66)	
**Sex, n (%)**			20,641
Male	10,797 (54)	1.08 (1.06-1.11)	
Female	9844 (46.3)	0.92 (0.90-0.95)	
Missing	603 (2.8)	—	
**Socioprofessional category, n (%)**			20,800
Retirees	6501 (31)	0.45 (0.44-0.47)	
Employees	5260 (25)	0.34 (0.33-0.36)	
Workmen	3300 (16)	0.19 (0.18-0.20)	
Managers, high intellectual professions	2708 (13)	0.15 (0.14-0.16)	
Craftsmen, shopkeepers, business leaders	1101 (5.3)	0.06 (0.05-0.07)	
Intermediate professions	1015 (4.9)	0.05 (0.05-0.06)	
No professional activity	895 (4.3)	0.04 (0.04-0.05)	
Farmer holders	20 (<0.1)	0.06 (0.06-0.15)	
Missing	444 (2)	—	
**Family status, n (%)**			19,837
Married or in couple	14,293 (72)	2.57 (2.50-2.65)	
Single	2604 (13)	0.15 (0.14-0.16)	
**Children**			
Yes, n (%)	15,611 (78)	3.54 (3.44-3.64)	19,987
Number, mean (SD)	2.13 (1.00)	—	13,923
Missing, n (%)	7321 (34)	—	—

a OR: odds ratio.

b Not applicable.

### Sociodemographic Analysis

In addition to age differences, significant variations in smartphone and social media use patterns were observed across sociodemographic groups. For example, socioprofessional categories such as managers and high intellectual professionals reported higher rates of smartphone use for work-related purposes (1,570 out of 2,708; 58.0%) compared to retirees (1,625 out of 6,501; 25.0%) and employees (2,104 out of 5,260; 40.0%), who predominantly used smartphones for entertainment. Similarly, participants with higher education levels (university graduates) demonstrated a greater prevalence of compulsive smartphone use (4,320 out of 6,000; 72.0%) compared to those with secondary education or less (5,490 out of 9,000; 61.0%). These findings underline the complex interplay between demographic factors and digital behaviors.

### Smartphone Compulsion Scores

Among the participants, 19,103 answered the SCT. A total of 8025 of 12,034 (66.7%) participants (odds ratio [OR] 2, 95% CI 1.90-2.11) exhibited compulsive smartphone use, and 7,020 (36,7%; OR 0.61, 0.58-0.64) scored ≥8, indicating obvious behavioral addiction (Table 2).

**Table 2.** Impact of smartphone use or problematic use.

Characteristics	Values, n (%)	OR^a^ (95% CI)	Answers, n
**Driving**			12,953
Smartphone use while driving (calls, SMS text messages, and GPS)	5736 (44)	0.37 (0.36-0.38)	
GPS set-up while driving	5577 (43)	0.36 (0.35-0.37)	
Sending messages while driving	1817 (14)	0.09 (0.09-0.10)	
Glancing at notifications while driving	4288 (33)	0.25 (0.24-0.26)	
**Nomophobia**			
Anxiety without smartphone	5582 (41)	0.36 (0.35-0.37)	13,660
Smartphone is a mental USB key	4527 (33)	0.27 (0.26-0.28)	13,636
Anxiety if smartphone discharge	3725 (27)	0.36 (0.35-0.37)	13,704
Anxiety if no network	3248 (24)	0.18 (0.17-0.19)	13,663
Smartphone malfunction as worrying as a child disease	2629 (19)	0.14 (0.14-0.15)	13,627
Find socket and have Wi-Fi as priority	1991 (15)	0.10 (0.10-0.11)	13,688
Anxiety if not able to answer call or notification	2036 (15)	0.11 (0.10-0.11)	13,651
No repair of broken screen for fear of separation	1261 (9.2)	0.06 (0.06-0.07)	13,642
Not lending own’s charger	1873 (14)	0.10 (0.09-0.10)	13,613
Sleep disorder if smartphone not near	1063 (7.8)	0.05 (0.05-0.06)	13,596
Athazagoraphobia	940 (7.3)	0.05 (0.04-0.05)	12,865
Phubbing	1818 (14)	0.05 (0.04-0.05)	12,865
Smombie	1452 (11)	0.09 (0.09-0.10)	12,865

a OR: odds ratio.

The percentage of participants according to the total number of positive responses by age class is displayed in Table 3.

**Table 3.** Smartphone Compulsion Test.

Total number of positive responses	18‐39 years, n (%)	40‐59 years, n (%)	≥60 years, n (%)
0	35 (0.8)	268 (3.5)	352 (4.6)
1‐2	338 (7.7)	842 (11)	766 (10)
3‐4	923 (21)	1301 (17)	1762 (23)
5‐7	1362 (31)	2066 (27)	2068 (27)
≥8	2504 (57)	2907 (38)	1609 (21)

The highest scores were seen in the youngest class of age: 57% (2504/4394) of those less than 40 years had a score of 8 or more. Around 45% of participants spent 1‐2 hours of their leisure time on screens, 7659 of 21,244 (36.1%) participants acknowledged going often or always to the toilets with their smartphone, and 9136 (43%) going to bed with it, although only 3186 (15%) said they fell asleep with it (Table 4).

**Table 4.** Median compulsion score according to activities with digital devices.

Characteristic	Median score (IQR)	*P* value
**Daily leisure time on screens**		.001^a^
≤25 minutes (n=936)	2 (1-4)	
26‐45 minutes (n=2086)	4 (2-6)	
46 minutes to 1 hour 30 minutes (n=2840)	6 (4-9)	
1 hour 31 minutes to 2 hours 15 minutes (n=2542)	7 (5-9)	
2 hours 16 minutes to 3 hours (n=1721)	8 (6-10)	
>3 hours (n=1827)	9 (7-12)	
**Going to the toilets with smartphone or tablet**		.001^a^
Never (n=3446)	4 (2-7)	
Rarely (n=3163)	6 (4-9)	
Often (n=3498)	7 (5-10)	
Always (n=1841)	9 (7-12)	
**Going to bed with a smartphone or tablet**		.001^a^
Never (n=3632)	4 (2-6)	
Rarely (n=1786)	4 (2-7)	
Often (n=2147)	7 (5-9)	
Always (n=4372)	8 (6-11)	
**Falling asleep with a smartphone or tablet**		.001^b^
Yes (n=2317)	9 (6-12)	
No (n=9784)	6 (4-9)	
**Consulting smartphone or tablet at wake-up**		.001^b^
Yes (n=7233)	8 (6-11)	
No (n=4682)	4 (2-7)	

a Kruskal-Wallis test.

b Mann-Whitney-Wilcoxon test.

### Problematic Smartphone Behaviors

Problematic smartphone use is displayed in Table 5.

**Table 5.** Median compulsion score according to smartphone use or overuse.

Characteristic		Median score (IQR)	*P *value^a^
**Smartphone and driving**			
**Smartphone use while driving (calls, SMS text messages, and GPS)**			.001
Yes (n=5350)		8 (2-5)	
No (n=6503)		5 (3-7)	
** GPS set-up while driving**			.001
Yes (n=5156)		7 (5-9)	
No (n=6697)		5 (3-7)	
**Sending messages while driving**			.001
Yes (n=1706)		9 (7-10)	
No (n=10,147)		6 (4-8)	
**Glancing notifications while driving**			.001
Yes (n=4000)		8 (6-10)	
No (n=7853)		5 (3-7)	
**Nomophobia**			
**Anxiety without smartphone**			.001
Yes (n=4972)		8 (3-7)	
No (n=6970)		5 (4-7)	
** Smartphone is a mental USB key**			.001
Yes (n=4120)		8 (6-9)	
No (n=7807)		5 (3-7)	
**Anxiety if smartphone discharge**			.001
Yes (n=3272)		9 (5)	
No (n=8705)		5 (5)	
**Anxiety if no network**			.001
Yes (n=2842)		9 (5)	
No (n=9106)		6 (5)	
**Smartphone malfunction as worrying as a child disease**			.001
Yes (n=2286)		8 (6)	
No (n=9623)		6 (6)	
** Find socket and have Wi-Fi as priority**			.001
Yes (n=1713)		8 (5)	
No (n=10,247)		6 (6)	
**Anxiety if not able to answer call or notification**			.001
Yes (n=1779)		9 (5)	
No (n=10,152)		6 (5)	
**No repair of the broken screen for fear of separation**			.001
Yes (n=1614)		8 (6)	
No (n=10,293)		6 (6)	
**Not lending own’s charger**			.001
Yes (n=1614)		8 (6)	
No (n=10,293)		6 (6)	
**Sleep disorder if smartphone not near**			.001
Yes (n=944)		11 (5)	
No (n=10,945)		6 (6)	
**Athazagoraphobia**			.001
Yes (n=862)		10 (5)	
No (n=10,914)		6 (6)	
**Phubbing**			.001
Yes (n=1688)		10 (5)	
No (n=10,088)		6 (5)	
**Smombie**			.001
Yes (n=1354)		10 (5)	
No (n=10,422)		6 (5)	

a Mann-Whitney-Wilcoxon test.

Unsafe driving habits were reported by less than half of participants, with 5736 of 12,953 (44.3%) participants admitting to using their smartphone while driving. A total of 1817 (14%) participants reported sending messages while driving, and 4288 (33.1%) acknowledged glancing at notifications while driving. Signs of nomophobia were common, with 5582 of 13,660 (40.9%) participants reporting anxiety without their smartphone. Phubbing was identified in 1818 (14.1%) participants, while 1452 (11.3%) described themselves as “smombies.”

### Social Media Use Patterns

Table 6 summarizes the answers about social networks.

**Table 6.** Social networks use.

Characteristics	Values, n (%)	OR^a^ (95% CI)	Answers, n
Use of social networks	12,102 (57)	1.33 (1.31-1.36)	21,244
**Type of social network**			12,196
Facebook	10,873 (89)	8.09 (7.85-8.33)	
WhatsApp	8166 (67)	4.49 (4.34-4.65)	
Instagram	5573 (46)	1.28 (1.23-1.34)	
Snapchat	3548 (29)	1.28 (1.23-1.34)	
LinkedIn	3258 (27)	1.02 (0.98-1.07)	
TikTok	2047 (17)	0.51 (0.49-0.54)	
**Network behavior**			
Log-in to simply watch without comments	8412 (70)	5.15 (4.99-5.32)	12,025
Scrolling through newsfeed without paying attention	6916 (57)	3.39 (3.28-3.51)	12,070
Too much time on social network	4800 (40)	1.96 (1.89-2.04)	12,143
Constant log-in to comment, post or like	2186 (18)	0.71 (0.68-0.75)	12,102
Log-in to simply watch without comments, and sadness afterward	1184 (10)	0.34 (0.32-0.36)	11,756
**Irrepressible need to connect more and more**			4787
Never	531 (11)	0.12 (0.11-0.13)	
Rarely	1321 (28)	0.39 (0.37-0.41)	
Often	2318 (48)	0.92 (0.88-0.96)	
Always	617 (13)	0.17 (0.16-0.19)	
**Unsuccessful tries to reduce use**			4752
Never	1293 (27)	0.36 (0.34-0.38)	
Rarely	1612 (34)	0.52 (0.50-0.55)	
Often	1527 (32)	0.47 (0.45-0.50)	
Always	320 (6.7)	0.07 (0.06-0.08)	
**Use with a negative impact on work or schooling or personal life**			4719
Never	2346 (50)	1.00 (—^b^)	
Rarely	1494 (32)	0.47 (0.45-0.50)	
Often	682 (14)	0.21 (0.19-0.22)	
Always	197 (4.2)	0.06 (0.05-0.07)	
**Overtime for thinking or planning next connection**			4774
Never	2777 (58)	1.00 (—)	
Rarely	1418 (30)	0.45 (0.43-0.48)	
Often	440 (9.2)	0.11 (0.10-0.12)	
Always	139 (2.9)	0.03 (0.03-0.04)	
**Agitation or upset if network access denied**			4771
Never	3444 (72)	1.00 (—)	
Rarely	947 (20)	0.27 (0.26-0.29)	
Often	265 (5.6)	0.06 (0.05-0.07)	
Always	115 (2.4)	0.02 (0.02-0.03)	

a OR: odds ratio.

b Not applicable.

A total of 12,102 (57%) participants (95% CI 56.0‐58.0) used social networks, primarily Facebook. Logging in to simply watch, without making comments, was reported by 8412 (70%) participants (OR 5.15, 95% CI 4.99-5.32), and 6916 (57%) scrolled through news feeds without paying attention. However, 4800 (40%) participants thought they spent too much time on social media. Questions relative to the problematic use of social networks were answered by fewer participants and generally acknowledged as not being a significant problem.

## Discussion

### Summary of Key Findings

Our EVADD study offers valuable insights into the various characteristics associated with frequent smartphone use, which has become a cornerstone of multitasking activities in modern life. By examining the problematic and potentially addictive aspects of this trend, we surveyed a significant sample of 21,244 adults in France, with an average age of 53 (SD 15) years, of whom 48% (n=9844) were female participants. This study revealed a high prevalence of problematic smartphone and social media use among French adults, with 8025 of 12,034 (66.7%) participants exhibiting compulsive behaviors and 4569 (38%) showing signs of addiction. These behaviors were more pronounced in younger age groups and were associated with activities such as unsafe driving and nighttime smartphone use. This work stands as a pioneering effort in France, shedding light on a topic of growing concern. The questions on problematic social media use were adapted from a combination of existing clinical frameworks and insights from the international literature. These were further refined through expert consensus to capture the primary characteristics of problematic social media behaviors. However, we acknowledge that these questions have not yet undergone formal validation.

Our findings underscore the widespread dependence on smartphones, with over two-thirds of participants exhibiting compulsive behaviors and a substantial portion devoting their free time to screen-based activities. While smartphones offer undeniable benefits for communication and information access, these patterns highlight the clinical and societal concerns associated with excessive use [11].

These findings should be viewed within the current post–COVID-19 landscape, where recent research indicates that digital behaviors have intensified and diversified, partly due to remote work, digital learning, and a broader shift toward digital entertainment and communication [8-10]. Such trends suggest that the patterns we observed may not merely reflect an exceptional period but rather a continued adaptation to new digital norms. The COVID-19 lockdown further exacerbated concerns about screen time, particularly among children, as evidenced by a study conducted by the French Public Health Agency. Almost 25% of respondents reported spending 7 hours a day, or even longer, on screens. Increased screen time, especially among young, educated individuals and urban dwellers, underscores the need for a nuanced understanding of its impact on physical and psychological well-being. However, it is essential to note that while screen time is a factor, its correlation with problematic smartphone use remains inconclusive [12].

### Clinical and Public Health Implications

Screen exposure has increased substantially, with nearly half of the participants dedicating over 1.5 hours daily to digital devices, reflecting a marked shift in leisure patterns. These findings align with broader societal trends observed during the pandemic. Parental concern regarding children’s screen time has escalated, a sentiment echoed by a study from the French Public Health Agency. This study highlighted a marked rise in average screen time during the COVID-19 pandemic, with nearly a quarter of the participants reporting screen engagement for 7 hours or more per day. This increase was predominantly observed among the youths, individuals with higher education levels, those working from home during the lockdown, and residents of urban areas [13]. Furthermore, a recent investigation indicated that individuals with a pronounced inclination toward smartphone use exhibit a higher propensity to consume video content on their devices, thereby accruing more screen time compared to their less engaged counterparts [14].

It is crucial to acknowledge that screen time is one of the several factors to consider in the discourse on problematic or excessive smartphone use. The current body of evidence regarding its impact on the physical and psychological well-being of young individuals remains mixed. While this study focuses on adults, we have included references to research on adolescents and children for several reasons. Adolescent populations have been the subject of most existing research on digital behaviors, offering critical insights into the psychological and behavioral consequences of smartphone use. These behaviors often persist into adulthood, making this research relevant for understanding patterns observed in adult populations. Additionally, intergenerational influences, such as how parents’ and children’s digital habits affect one another, further justify the inclusion of this literature. Addressing findings from adolescent-focused studies also enables us to draw attention to the life-course perspective of problematic smartphone use and its long-term implications. Recent comprehensive reviews and meta-analyses have found no significant link between screen time that surpasses the American Academy of Pediatrics guidelines and adverse health outcomes in youths [15]. The adage “everything in moderation” arguably serves as pragmatic counsel for parents navigating discussions on screen time with their children. However, the efficacy of screen time regulation as a strategy to curb problematic behaviors among youths does not consistently align with this principle [16].

Based on our findings, we propose the following actionable recommendations:

- For policy makers: Develop and implement public awareness campaigns that emphasize the risks of excessive smartphone use, particularly among younger adults. Consider incorporating educational modules on digital well-being in school curricula.
- For mental health professionals: Integrate screening for problematic smartphone use into routine mental health assessments, particularly for individuals reporting anxiety, sleep disturbances, or compulsive behaviors. Provide targeted interventions, such as mindfulness-based stress reduction, sophrology, or therapeutic yoga, to address underlying factors contributing to excessive smartphone use. These approaches have demonstrated efficacy in reducing compulsive behaviors, improving emotional regulation, and promoting digital well-being.
- For employers: Encourage balanced digital use among employees by promoting “tech-free” breaks and fostering a culture that prioritizes mental well-being over constant connectivity.

These recommendations aim to support a multistakeholder approach to mitigating the health and social risks associated with problematic smartphone use.

### Impact on Habits and Behaviors

Our investigation shed light on notable shifts in habitual behaviors, particularly concerning smartphone use in various contexts. A significant finding was that 36% of participants reported frequent or constant smartphone use in the bathroom. Beyond the hygiene concerns and the potential for bacterial contamination associated with this practice, it has emerged as a compulsive behavior for some individuals [17]. A survey from the United Kingdom disclosed that 57% of respondents confessed to using their phones in the bathroom, with 8% doing so “always” and an additional 14% “most of the time” [15]. In France, a study conducted in September 2019 with a representative sample of 1024 individuals found that 46% bring their mobile phone or tablet into the bathroom [18]. Younger participants exhibited significantly higher rates of compulsive smartphone use, likely driven by greater reliance on social media and constant connectivity. These findings highlight the need for targeted interventions addressing age-specific patterns of use. Furthermore, socioprofessional categories displayed distinct patterns: managers and high intellectual professionals reported higher screen time dedicated to work-related activities, while retirees and employees showed greater use of digital devices for entertainment and social purposes. These differences emphasize the importance of designing tailored public health interventions to address the specific risks and needs of each group. The sociodemographic differences observed in smartphone and social media use patterns provide important insights for tailoring public health interventions. For instance, the higher prevalence of compulsive smartphone use among individuals with advanced education and socioprofessional categories suggests the influence of work-related pressures and digital connectivity on these behaviors. In contrast, retirees and employees primarily using smartphones for entertainment highlight a different set of needs and risks. These distinctions emphasize the necessity of demographic-specific strategies to address problematic smartphone use effectively.

The intersection between smartphone use and sleep disturbance warrants close examination. Research in this area has predominantly focused on adolescents and young adults [19]. Our results reveal that smartphone engagement prior to sleep, including exposure to bright blue light and the use of applications, adversely affects sleep quality. A notable 43% of participants indicated that they go to bed accompanied by their smartphone or tablet, with 15% falling asleep while still engaging with these devices. Additionally, 53% reported awakening alongside their phones. Exposure to blue light emitted by smartphones during evening hours can inhibit melatonin synthesis, disrupt circadian rhythms, and reduce total sleep duration [20]. “Doomscrolling” further exacerbates these issues, delaying sleep onset and increasing daytime fatigue. Nighttime social media use, particularly when emotionally engaging with content, is linked to poorer sleep quality [20].

Our findings reveal concerning behaviors: 44% of respondents conceded to using their smartphones while operating a vehicle, with 14% engaging in SMS text messaging and 33% reading messages. It is important to note, however, that this figure includes the use of smartphones for GPS navigation. When the phone is securely mounted and not manipulated during driving, this use may not constitute a dangerous behavior and is more akin to traditional GPS device use. Such practices not only compromise the safety of the individuals involved but also pose significant risks to public safety [21]. A study elucidated that the propensity for distraction during driving is prevalent, particularly among drivers below 50 years of age [22]. Insights from the French Road Safety Observatory highlight that smartphone use while driving amplifies accident risks 3-fold and increases the likelihood of accidents by 23 times when reading messages [23].

### Strengths and Limitations

Our cohort, while substantial, does not fully represent the diversity of the French population due to its size and the uniformity of its participants. The reliance on a nonprobability sample limits the generalizability of the findings. While the large sample size ensures statistical power, the respondents may not be representative of the broader French population, particularly in terms of socioeconomic and cultural diversity. Future studies should consider probability sampling methods to address this limitation and enhance external validity.

While this study captures essential behaviors through adapted clinical frameworks, the lack of validated tools in French limits generalizability, highlighting the need for future validation studies. Additionally, a limitation of this study is the reliance on several single-item measures to assess specific behaviors, such as smartphone use during nighttime or while driving. Although these measures provide valuable insights, they may lack the reliability and depth of multi-item scales. Future research should aim to develop and validate more comprehensive tools to assess these constructs with greater precision and reliability.

The study focused on adults, excluding children and adolescents. Dissemination via a private internet link may have introduced selection bias, potentially affecting participation among less technology-savvy individuals. Additionally, individuals with a pre-existing interest in digital technologies may have been overrepresented in the sample.

The choice of nonparametric methods was driven by the observed nonnormal distribution of the data, as indicated by the Shapiro-Wilk test. These methods ensure robust analysis for ordinal and nonnormally distributed data but may limit the generalizability of the findings compared to parametric approaches. Future research with larger and more representative datasets could explore additional statistical methods to further validate these results. While the study provides valuable insights into demographic differences in digital technology use, further research is required to examine cultural, psychological, and contextual factors influencing these behaviors across diverse populations.

A limitation of this study is the lack of validated tools in French for assessing problematic smartphone and social media use. While the SCT has been widely used in clinical practice and provides a broad evaluation of problematic smartphone behaviors, its psychometric properties require formal validation. Similarly, the questions on problematic social media use were adapted from a combination of existing clinical frameworks and insights from the international literature. These were further refined through expert consensus to capture the primary characteristics of problematic social media behaviors. However, we acknowledge that these questions have not yet undergone formal validation. Future research should prioritize the psychometric validation of these tools to ensure their reliability and generalizability across diverse populations.

A key challenge in the study of smartphone and digital technology use is the proliferation of overlapping constructs and terminologies, which can lead to fragmentation in the field. Terms such as problematic smartphone use, smartphone addiction, and compulsive smartphone use often describe similar phenomena. Similarly, behaviors like FoMO and specific manifestations such as phantom ringing or notification behavior overlap with broader constructs like nomophobia. Standardization of terminology and constructs in future research will be essential for advancing understanding and developing unified measures.

### Future Directions

The EVADD study provides an epidemiological and clinical perspective on smartphone use and its consequences within an adult demographic aged 18 to 60 years. The impact on older populations remains largely unexplored [24]. In light of the growing concern over digital device use, the French government recently announced the commissioning of an expert report to address appropriate screen use in domestic and educational settings. Concurrently, the Canadian Paediatrics Society Working Group has highlighted several potential research paths [25].

## Abbreviations

CHERRIES: Checklist for Reporting Results of Internet E-Surveys
CROSS: Checklist for Reporting of Survey Studies
EVADD: Evaluation of Digital Addiction
FoMO: fear of missing out
OR: odds ratio
SCT: Smartphone Compulsion Test
